# Study on the Perception Mechanism of Utricles Based on Bionic Models

**DOI:** 10.3390/biomimetics7010028

**Published:** 2022-02-23

**Authors:** Yani Jiang, Xianjin Wang, Shien Lu, Yongbin Qin, Can He, Yixiang Bian

**Affiliations:** School of Mechanical Engineering, Yangzhou University, Yangzhou 225000, China; ynjiang@yzu.edu.cn (Y.J.); huyue0131@163.com (X.W.); huhu1232022@163.com (S.L.); qfen1010@126.com (Y.Q.); xwen11111@126.com (C.H.)

**Keywords:** bionic macula (BM), sensory cell, surface symmetrical electrodes PVDF (polyvinylidene difluoride) fiber with metal core (SMPF) sensor, bionic macula with sand (BMS), bionic utricle (BU)

## Abstract

Background: The relationship between utricle diseases and structural lesions is not very clear in the clinic due to the complexity and delicacy of the utricle structure. Therefore, it is necessary to study the perception mechanism of the utricle. Methods: Imitating the sensory cells in the macula of the utricle, a symmetrical metal core PVDF fiber (SMPF) was designed as a bionic hair sensor to fabricate a bionic macula (BM), a bionic macula with sand (BMS) and a bionic utricle (BU). Then experiments were carried out on them. Results: This indicated the SMPF sensor can sense its bending deformation, which was similar to the sensory cell. The amplitude of the output charges of the SMPF in BMS and BU were significantly improved. The SMPF, whose electrode boundary was perpendicular to the impact direction, exhibited the largest output charges. Conclusion: The presence of otoliths and endolymph can improve the sensing ability of the utricle. The human brain can judge the direction of head linear accelerations based on the location of the sensory cell in the macula that produces the largest nerve signals. This provides a possibility of studying utricle abnormal functions in vitro in the future.

## 1. Introduction

The inner ear (vestibular system) on one side of the human head is composed of three semicircular canals and otoliths (utricle and saccule) [1]. The semicircular canals can sense head angular accelerations, while the utricle and saccule can sense head linear accelerations [2,3]. There is a macula at the bottom of the utricle and the anterior upper wall of the saccule. Sensory cells and connective tissue are located at the bottom of the macula, and a glial membrane covers the sensory cells [4]. The cilia of the sensory cells extends into the glial membrane. Sensory cells exhibit different polarization directions due to the different arrangements of the cilia. Small otoliths (calcium carbonate particles) are embedded above the glial membrane [5]. The utricle and saccule are full of endolymph. A strip-shaped region, which is called the striola, is located on the surface of the macula. The polarization directions of the sensory cells on two sides of the striola are opposite [6]. When the head moved, the sensory hair cells in the utricle were deformed mainly due to the inertial force of the macula [7]. Further, this can trigger a mechanical-electrical conduction reaction of the sensory cells and induce corresponding neural signals [8]. The brain can perceive the linear acceleration of the head when it receives these neural signals [9]. Utricles play a very important role in human spatial positioning, visual gazing, and balance [10].

At present, the relationship between utricle diseases and structural lesions or functional abnormalities is not very clear in the clinic [11]. To deeply understand the working principle of the complex and subtle otoliths and accurately judge the specific causes and locations of abnormal utricle function, it is necessary to understand the detailed perception mechanism of the utricle. Skalak et al. established a classical utricle elastic model for the first time [12]. In this model, the endolymph, otolithic membrane, and glial layer were regarded as a Newtonian liquid, rigid body, and elastomer, respectively, and dynamic responses of the macula were given. Based on the classical elastic model, Grant et al. regarded the glial layer as a ‘Kelvin-Voigt’ viscoelastic material and proposed numerical results of the dynamic response of the utricle [13]. Goldberg et al. established a simplified utricle sensing model [14]. They simplified the utricle into a sensing element, which could sense the tilt angles of the head, the sudden impacts applied to the head, and the response of head linear acceleration [15]. Burgess et al. used ocular vestibular evoked myogenic potential (oVEMP) to study the sensing function of the otolith organ, showing that it can sense the linear vibration of the head, including parameters, such as waveform, frequency, and gain [16]. Grant et al. also used oVEMP to study the perception function of the utricle under the low-frequency vibration [17].

The above typical biomechanical models have greatly promoted the study of the relationship between the structure and function of the human utricle [18]. However, due to its complex structure, small volume, and deep burial location in the head, it is difficult to directly reveal the mechanical response of the utricle by existing technical means, such as clinical images. To date, the sensing mechanism of the utricle to linear acceleration, that is, the sensing process of the utricle to the specific direction and amplitude of the head linear acceleration is uncertain. To our knowledge, the effect of the shape of the striola has not been studied clearly. These factors restrict people’s understanding and treatments of some utricle diseases [19].

Physical models fabricated using artificial materials (or devices) to imitate biological tissues in human physiological otoliths exhibit many advantages [20], such as easy to observe experiments, easy to carry out experiments with ethical restrictions, and easy to promote our understanding of the causes of some related vestibular diseases [21]. Gong and Wang designed an artificial vestibular model with a built-in sensor [20,22]. Imitating the function of the human physiological otolith, the model was prepared using a uniaxial piezoelectric ceramic vibration gyroscope to sense acceleration. Santina et al. prepared a first-generation artificial vestibular system, MVP (Multichannel Vestibular Prosthesis)1 [23]. In their model, three mutually perpendicular micro gyroscopes were used as sensors to detect the angular accelerations around three axes. Based on these studies, Chiang et al. fabricated a smaller second-generation artificial vestibular system, MPV2, using a yaw axis angular velocity sensor and a biaxial pitch gyroscope to detect acceleration [24].

In the above artificial vestibular systems, the devices to detect head velocities and accelerations were all solid inertial element sensors. Their structures and working principles were completely different from those of human physiological otoliths; thus, they were not very helpful in researching the vestibular system [25]. In the human physiological utricle, the sensing organs are sensory cells. Therefore, sensors with structures and sensing mechanisms similar to those of sensory hair cells can better imitate the human utricle. Hu and Liu designed a piezoresistive artificial hair sensor and installed it in a pipeline [26]. In their study, the bending of the hair cantilever caused by the liquid flow was sensed through the change of the resistance value at the fixed end, and the velocity and acceleration of the pipeline were obtained indirectly by calculating the flow velocity of the liquid. However, the geometric structure of the model was completely different from that of the human utricle [27,28].

In this paper, a surface symmetrical electrode PVDF (polyvinylidene difluoride) fiber with a metal core (SMPF) sensor was prepared by imitating the structure and sensing function of sensory cells in a human utricle, its sensing function was verified, and a mathematical model of its sensing process was established. Furthermore, according to the semicircular arc shape of the human utricle micro pattern, multiple SMPFs were implanted into silicone rubber to prepare bionic macula (BM). According to the structure of the human physiological utricle, the BM is fixed at the corresponding part of the inner wall of the resin shell and filled with liquid to prepare a bionic utricle (BU) with a similar structure and function to the human utricle. Experiments are carried out on the BM and BU, and the sensing mechanism of the acceleration model is studied through the output charges of the SMPFs. In addition, the effects of otoliths and the endolymph in the utricle on the perception of the linear acceleration of BU were studied. In particular, the mechanism of action of the striola has been studied in depth. Based on the results obtained, the perception mechanism of the human utricle and the influence of structure on function are explored and inferred.

## 2. Fabrication of an SMPF Sensor

**Figure 1:**
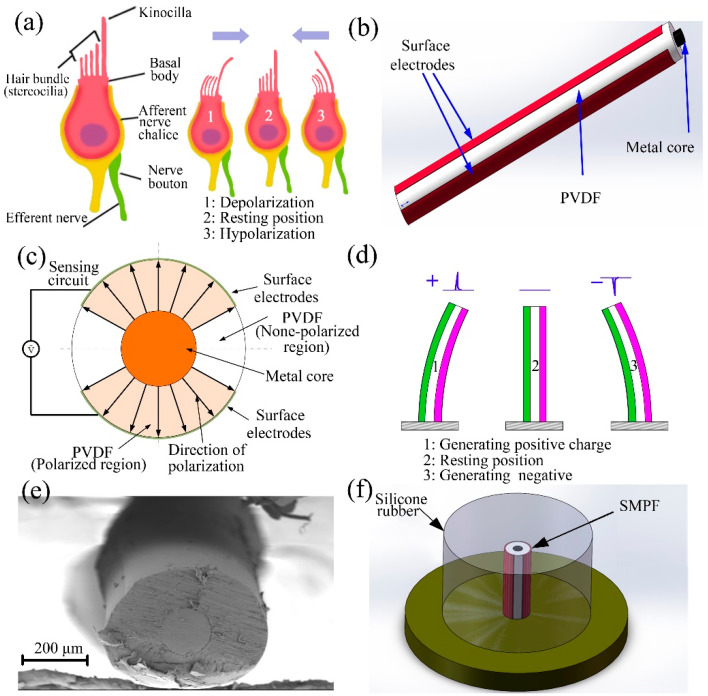
(**a**) Diagrams of the sensory cell and its sensing function for deformation. (**b**) Diagram of the SMPF structure. (**c**) The cross-section of the SMPF. (**d**) The sensing function for deformation. (**e**) A SEM image of the SMPF. (**f**) A diagram of the SMPF-R.

**Figure 2:**
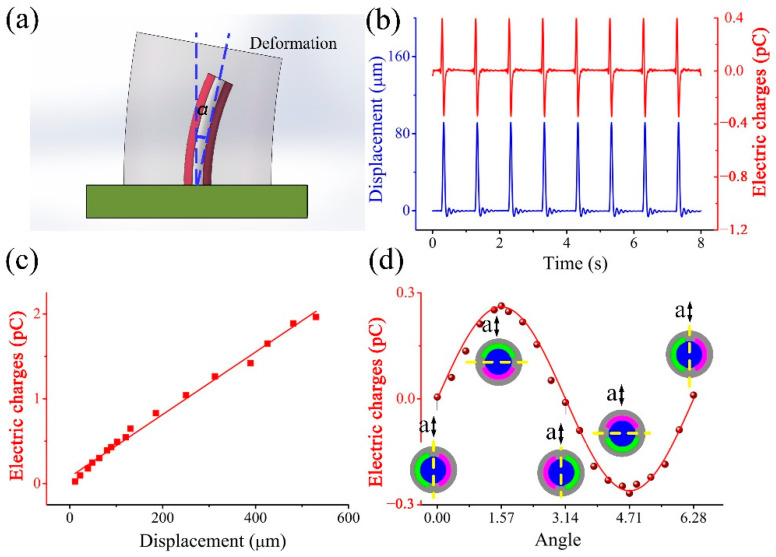
(**a**) A diagram of the experimental system. (**b**) Transverse deformation displacement and output charges of the SMPF under impact oscillation. (**c**) The relation between the deformation amplitude of the SMPF sensor and its output charges. (**d**) The relation between the SMPF sensor output charges and the included angle between the impact oscillation direction and the electrode interface on the SMPF sensor surface.

Biosensors of the human utricle are mainly sensory cells located at the bottom of the macula. During head movement, the kinocilium, and stereocilia of the sensory cell are bent and deformed mainly due to the inertial force of the otoliths. When stereocilia bends toward kinocilia due to external pressure, it can lead to the depolarization of the sensory cells. Otherwise, it can lead to hyperpolarization of the sensory cell when the bending direction is opposite, as shown in Figure 1a [10]. An SMPF sensor was designed to mimic the structure and function of sensory cells. The process of fabrication of an SMPF sensor has been reported in our previous work [29]. Briefly, PVDF particles were put into a metal container and heated to the molten state and then extruded together with a metal wire from a small hole. After air cooling, a fiber embryo was obtained. Two thin metal layers with the same shape were coated on the symmetrical position of the surface of the fiber, and they were used as surface electrodes. After being cut to the required length, the fiber embryo was placed in hot silicone oil, and a DC voltage was applied on the metal wire and the surface electrodes to polarize the PVDF layer, and then an SMPF sensor can be fabricated. Diagrams of the structure and the polarization direction of the SMPF are shown in Figure 1b,c. The structure and function of the SMPF were similar to those of the sensory cells, and they both sensed the directions and amplitudes of their bending deformation. A diagram of the SMPF sensing function is shown in Figure 1d. When the SMPF bends, electric charges can be generated on the surface electrodes due to the piezoelectric effect of PVDF. The polarities of the charges depend on the bending direction of the SMPF. The amplitudes of the bending deformation can be obtained according to the amounts of charges. A SEM (scanning electron microscope) image of the SMPF is shown in Figure 1e. The metal core is evenly wrapped by a PVDF layer, and the diameter of an SMPF is approximately 300 μm, then a SMPF encapsulated with silicone rubber was obtained by embedding a single SMPF in silicone rubber (named SMPF-R), as shown in Figure 1f, and its sensing ability was verified. The SMPF-R was placed on a worktable and subjected to impact oscillation using an electromagnetic exciter, and its deformation was measured using a laser displacement sensor. The deformation of the SMPF-R is shown in Figure 2a. As shown in Figure 2b, the transverse displacement of the SMPF-R and the output charges of the SMPF sensor all exhibit an impact waveform, and their frequency and phase are the same. The SMPF sensor outputs not only positive charges but also negative charges, which are due to the inherent characteristics of the piezoelectric material. The amplitude of the electric charges of the SMPF sensor had a linear relationship with the amplitude of displacement of the SMPF-R, as shown in Figure 2c. The amplitude and polarity of the electric charges were changed along with the impact oscillation direction. The included angle between the impact oscillation direction and the electrode boundary on the SMPF sensor surface is regarded as *δ*. The charges are sinusoidal with the included angle, as shown in Equation (1). The specific derivation process is shown in the Supplement Materials.

(1) $$Q=Q_{0}\times U_{0}\times \sin\delta$$

where *Q* is the output electric charge of the SMPF sensor, *Q*_0_ is a parameter that is determined by the SMPF-R, and *U*_0_ is the amplitude of the impact oscillation.

The above results showed that the SMPF sensor could sense the direction, waveform, frequency, phase, and other parameters of the impact oscillation acting on the SMPF-R. Its sensing function was very similar to that of sensory cells in human macula [30]. Therefore, it could be concluded that the SMPF sensor was very suitable to be used to imitate sensory cells and to fabricate bionic macula (BM) and bionic utricle (BU).

## 3. Fabrication of a BM and Its Sensing Ability

**Figure 3:**
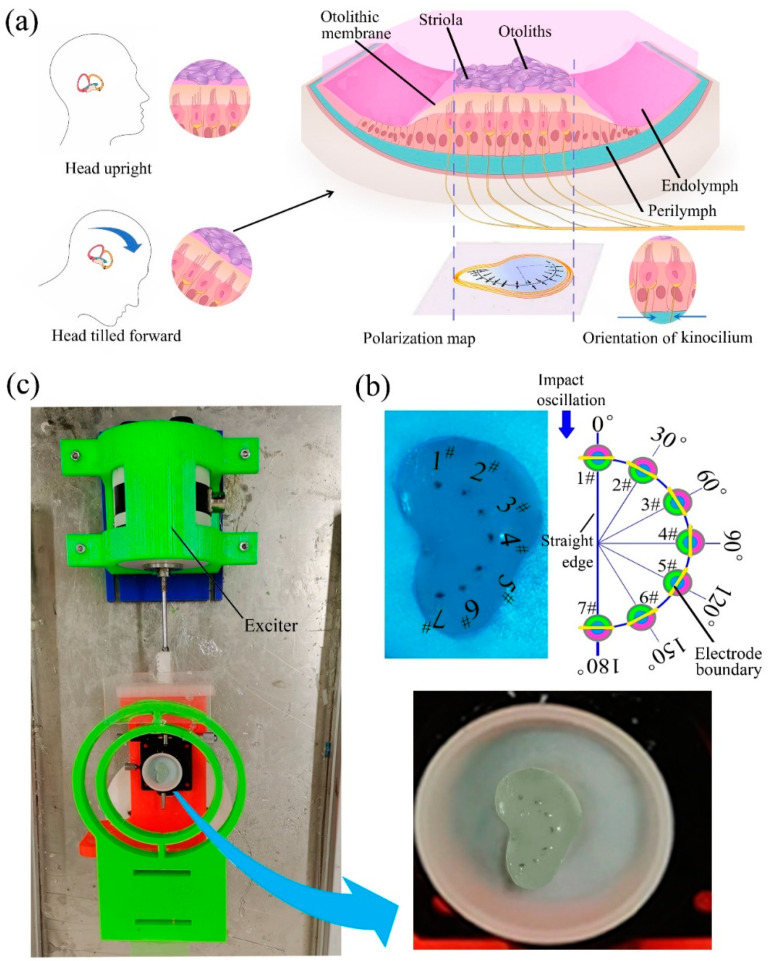
(**a**) A diagram of the human macula. The polarity of sensory cells on one side of the striola is opposite to that on the other side. (**b**) A photograph of the BM. (**c**) A photograph of the experimental system.

The structure of the utricle, shown in Figure 3a, uses sensory cells to perceive the linear acceleration of the head. On the surface of the macula, there is a semicircular arc-shaped striola. The sensory cells on opposite sides of the striola have opposite polarity. Based on the shape of the striola, the SMPF arrays were arranged and embedded in silicone rubber to fabricate a BM; then, a series of experiments were carried out on the BM to explore its sensing mechanism of linear acceleration.

**Figure 4:**
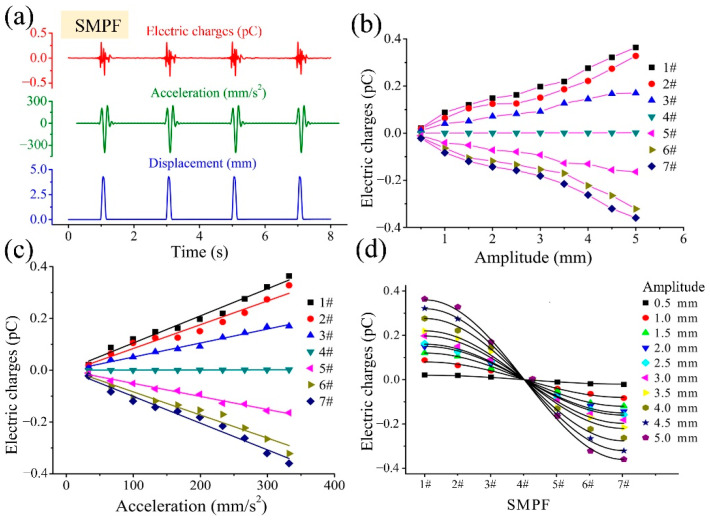
(**a**) The displacement and linear acceleration of the shell, as well as the output charges of SMPF1#. (**b**) The relation between the output charges of the seven SMPF sensors and the displacement amplitude of the shell. (**c**) The relation between the seven SMPF output charges and the shell linear acceleration. (**d**) The output charges of the seven SMPF sensors at different amplitudes of impact oscillation.

First, imitating the structure of the human utricle, the shell of the BU was designed and manufactured using the 3D printing method, which had a ratio of 10:1 to that of humans. A BM was designed and fabricated with silicone rubber using the pouring method. Seven SMPF sensors, numbered 1# to 7#, were embedded in silicone rubber along a curve similar to the striola arc and distributed evenly. The electrode boundary on the SMPF surface is tangent to the striola arc, and the central angle of the two adjacent SMPFs is 30°, as shown in Figure 3b. The BM was then subjected to impact oscillations in the direction parallel to the straight edge, as shown in Figure 3c. The waveforms of the transverse displacement and linear acceleration of the shell and the output charges of the SMPF1# sensor are shown in Figure 4a. The three waveforms exhibit the same frequencies. For the convenience of statistics and comparison, the first peak value in a single impact was taken as an amplitude. The output charge amplitudes of the seven SMPFs are shown in Figure 4b. The output charge amplitudes of the seven SMPFs increase with increasing impact amplitude, and they are quite different at the same deformation amplitude. This was because the surface electrode boundaries of the seven SMPFs were separately tangent to the semicircular arc. The relationships between the linear accelerations of the BM and the output charge amplitudes of the seven SMPFs are shown in Figure 4c. The output charge amplitudes of the seven SMPFs are linearly related to the linear acceleration of the BM, which indicates that the transverse deformations of the seven SMPFs were also linear with the linear acceleration. As shown in Figure 3g, subject to an impact oscillation with the same amplitude, the amplitudes of each SMPF exhibit a cosine distribution, which confirms Equation (2). It could be concluded that the seven SMPFs in BM could sense the waveform, frequency, phase, and amplitude of the impact oscillation acting on the BM. Based on these results, it is speculated that, in the human body, the deformation of the whole macula should be linear with the head linear acceleration.

## 4. Development of a BU and Its Sensing Mechanism

**Figure 5:**
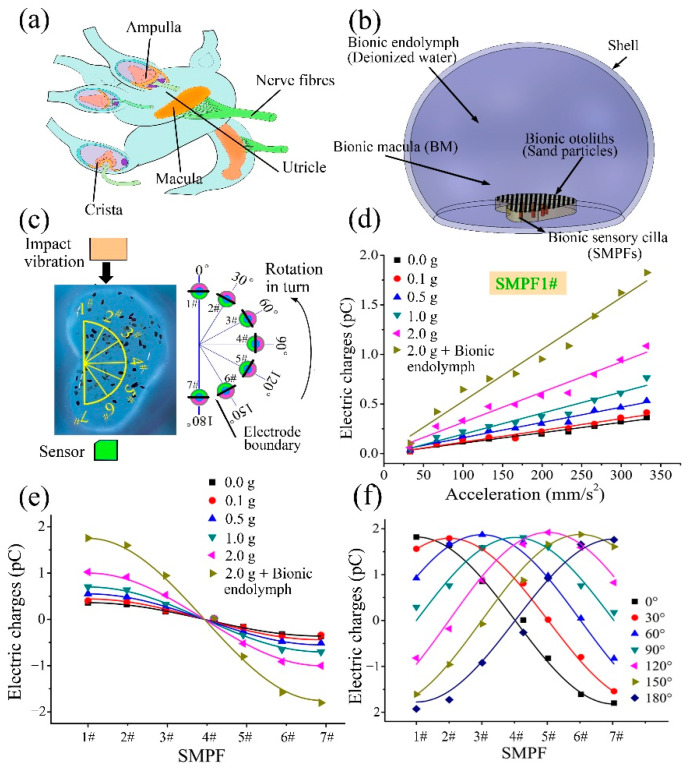
(**a**) A diagram of the structure of the human utricle. (**b**) A diagram of the BMS and the BU. (**c**) A photograph of the BMS and the experimental system. (**d**) The relation between the output charges of the SMPF1# sensor and the linear acceleration of the shell. (**e**) The output charges of the seven SMPF sensors at the same amplitude of impact oscillation. (**f**) The output charges of the seven SMPF sensors at different directions of impact oscillation.

In the human body, there are many otoliths on the otolith membrane of the utricle. The otoliths, which are mainly composed of protein and calcium carbonate, exhibit a higher density than that of surrounding tissue. Thus, the effect of otoliths on the perception of the utricle should not be ignored. Therefore, in this part, imitating the structure of the human utricle (as shown in Figure 5a), sand particles with different masses were embedded on the BM surface to fabricate a bionic macula with sand (BMS), which was installed at the bottom of the utricle shell, as shown in Figure 5c. Deionized water was injected into the shell to fabricate a bionic utricle (BU), as shown in Figure 5b. The BMS and BU were successively subjected to impact oscillation in the direction parallel to the straight edge, as shown in Figure 5c. Under impact oscillation, the waveform to the displacement of the shell of the BMS and the BU and the output electric charges of the SMPF sensors were similar to that of the BM shown in Figure 5d, but their amplitudes were significantly larger than that of the BM. The amplitude of the SMPF1# output charges with different impact accelerations is shown in Figure 5d. The amplitude of the output charges increases with increasing sand mass in the BMS under the same acceleration and is further improved in the BU. The amplitude of the output charges of SMPF 1# is linear with acceleration, which is similar to the experimental results of the BM in Figure 3f. However, the fitting line slope in Figure 5d is significantly larger than that in Figure 3f, which indicates that the sensitivity of the BMS and BU is larger than that of the BM. The BMS and the BU were then subjected to impact oscillation with the same amplitude, and the result is shown in Figure 5e. The amplitudes of the output charges of the SMPFs are distributed according to a cosine curve, which is similar to the result of the BM shown in Figure 3g. The filled endolymph increased the quantity of the output charges. This may be due to fluid inertia, which could help to increase the deformation of the SMPF sensor. Finally, as shown in Figure 3c, the BU around the striola arc was rotated so that the electrode boundary of each SMPF in it was successively perpendicular to the impact oscillation direction, and a diagram of this experiment is shown in Figure 4c. The results shown in Figure 5f indicate that in a single impact oscillation cycle, the amplitude of the output charges of the special SMPF, whose surface electrode boundary was perpendicular to the impact direction, was larger than those of the other SMPFs, and the amplitudes of the output charges of each SMPF at different positions exhibited a cosine distribution. Based on these experimental results, it can be speculated that in the human utricle, under head acceleration, otoliths could increase macular deformation due to their higher density, as could the endolymph, which can act as fluid inertia on the otolith membrane, which is consistent with that by Buskirk et al. [31]. Thus, the existence of otoliths and the endolymph can increase the neural signals of sensory cells and improve the ability of the utricle to perceive head accelerations. Most likely, the human brain determines the direction of the head linear acceleration by the location of the sensory cells along the striola that output the maximum neural signal and determines the magnitude of the head linear acceleration by the maximum neural signal.

Based on the above results in this paper, when an impact oscillation was applied to the BU, the output charges of the seven SMPF sensors can be described as follows:

(2) $$Q_{i}=A_{0}B_{0}\cos\left|(i-1)\times \frac{\pi }{6}-\gamma \right|$$

where *i* is the sequence number of the SMPF, *A*_0_ is the amplitude of the impact oscillation, *B*_0_ is a constant determined by some parameters, including masses of the silicone rubber, sand particles, and endolymph, viscosity of the endolymph, size of the SMPF, *γ* represents the included angle between the straight edge of the striola arc and the impact direction. It could be concluded from Equation (2) that when the impact oscillation direction was just perpendicular to the electrode boundary on the special SMPF surface, the amplitude of the SMPF output charges reached a maximum.

## 5. Discussion and Conclusions

In this paper, imitating the sensory cells in the human utricle, an SMPF sensor was developed. The experimental results showed that the SMPF sensor exhibited a similar function to sensory cells in the human utricle. Using seven SMPF sensors, a BU was then fabricated to imitate the human utricle. When the BU was subjected to an impact oscillation, the amplitude of the output electric charges of the SMPF sensors was linear with the amplitude of the impact oscillation. Due to the existence of sand particles and deionized water, the amplitude of the output charges of the SMPF in the BU was significantly improved. In an impact oscillation cycle, the amplitude of the output charges of the special SMPF, whose surface electrode boundary was perpendicular to the impact direction, was the largest. The output charge amplitudes of all SMPF sensors exhibited a cosine distribution.

Based on these results, it can be speculated that, in the human utricle, sensory cells can sense macular deformation and head linear acceleration. A single sensory cell can perceive the deformation direction or amplitude of the macula but does not perceive them at the same time. Therefore, many sensory cells are arranged along the arc-shaped striola, and the direction of the polarity of the sensory cells is perpendicular to the arc curve. When the head moves, the macula deforms due to the inertial force of the otoliths and the glial membrane and the friction force of the endolymph. When the polarization direction of the sensory cell is the same as the deformation direction of the macula, the neural signals generated by it are the largest. Therefore, according to the location of the sensory cell that generates the largest neural signal and its largest neural signal, the brain can determine the direction and amplitude of the deformation of the macula and then determine the direction and amplitude of the linear acceleration subjected to the head. Otoliths and the endolymph improve the deformation of the macula and enhance the perception of the utricle. The experimental results also show that the perception function of BU in this paper is consistent with the test results of humans [16,17].

## Data Availability

Not applicable.

## Supplementary Materials

The following are available online at https://www.mdpi.com/article/10.3390/biomimetics7010028/s1, Figure S1: Internal structure of the otoliths; Figure S2: The polarized direction of the otolith hair cells of the utricle; Figure S3: The shape of the striola and the distribution of the hair cells; Figure S4: The Schematic diagram of the structure of a SMPF; Figure S5: Polarizing circuit of an SMPF; Figure S6: Sensing circuit of an SMPF; Figure S7: The bending deformation of an SMPF; Figure S8: The BU subjected to an impact vibration [10,19,32].
