# Supplementary material for: Study on the Perception Mechanism of Utricles Based on Bionic Models
Source: Biomimetics (Basel). 2022 Feb 23;7(1):28. doi: 10.3390/biomimetics7010028 (PMC8945493; doi:10.3390/biomimetics7010028)
Supplement: Supplementary file 1 [file biomimetics-07-00028-s001.zip › biomimetics-1585672-supplementary.pdf]

Supplementary Materials

Study on the Perception Mechanism of Utricles Based on Bionic Models

Yani Jiang, Xianjin Wang, Shien Lu, Yongbin Qin, Can He, Yixiang Bian*

School of Mechanical Engineering, Yangzhou University
ynjiang@yzu.edu.cn (Y.J.); huyue0131@163.com (X.W.); huhu1232022@163.com (S.L.); qfen1010@126.com (Y.Q.); xwen11111@126.com (C.H.)
* Correspondence: yxbian@yzu.edu.cn

1. Structure and function of utricle and sensory cells

The otolith organ in the human inner ear, consisting of a utricle and a saccule. The outer shell of the utricle is irregularly spherical and filled with endolymph. There is a macula containing sensory cells in the bottom of the utricular shell. Some otoliths are located on the top of the macula. The mesh layer is below the otoconial layer. The supporting cells and hair cells are located at the bottom of the utricular shell. The cilia of the hair cells extend into gel layer. The structure of the macula is shown in Fig. S1[32].

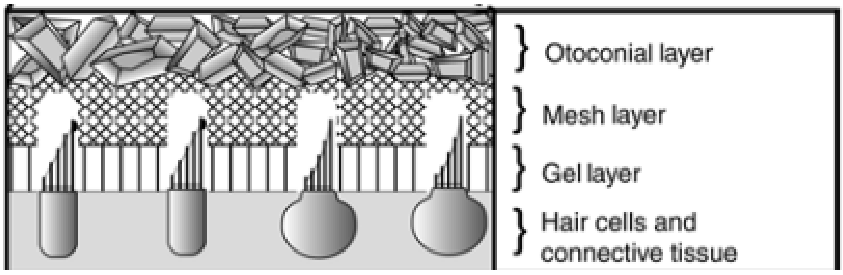

Figure S1 Internal structure of the otoliths

In the human utricle, a sensory hair cell has multiple stereocilia and one kinocilia on its top. When the cilia are deformed, the sensory cells will produce sensory signals to sense the deformation of the cilia. The polarization direction of the sensory cells is determined by the positional relationship between the stereocilium and the kinetocilia, as shown in Fig. S2[33]. Due to the existence of the polarization direction, when the cilia are subjected to an external force and deform at different directions, the cells will produce different sensing signals. This property enables the sensory cells to sense the deformation of the cilia in different directions.

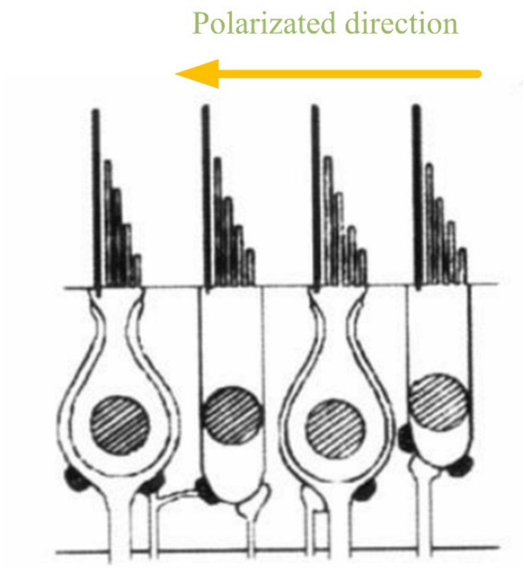

Figure S2 The polarized direction of the otolithic hair cells of the utricle

In the utricle, there is a semicircular striola on the surface of the macula. On each side of the striola, the sensory cells are arranged in opposite polarization directions, as shown in Fig. S3[34]. In addition, on both sides of the striola, the polarization of the sensory cells is perpendicular to the striola curve. When the transverse deformation of the macula occurs, for the "retractive-pulling" effect, the sensory cells at the opposite sides of the striola can generate opposite deformation and opposite signal.

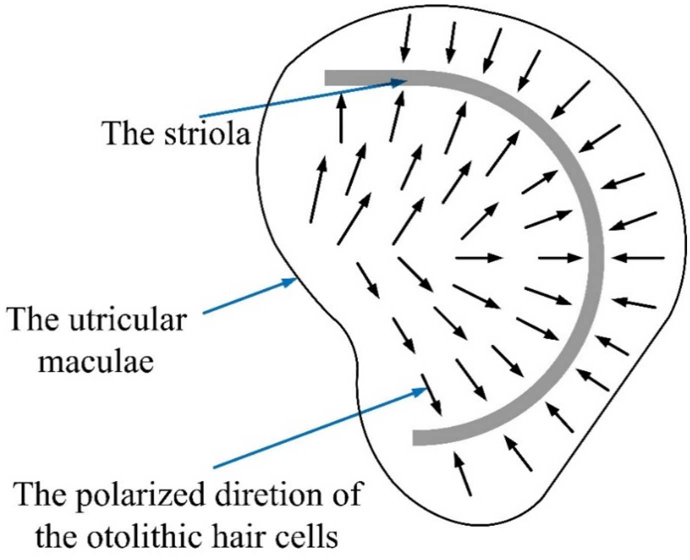

Figure S3 The shape of the striola and the distribution of the hair cells

2. Structure of SMPF

The structure of SMPF (Symmetric metal core PVDF fiber) is shown in Fig. S4. There is a metal core with a radius of R_m in the middle of the SMPF. The metal core is surrounded by a uniform thickness PVDF layer with a radius of R_c. Two symmetrical thin metal layer are coated on the surface of PVDF along the longitudinal direction, which are used as the surface electrodes. The angle of the coverage of the thin metal layer is α. The length of SMPF is L.

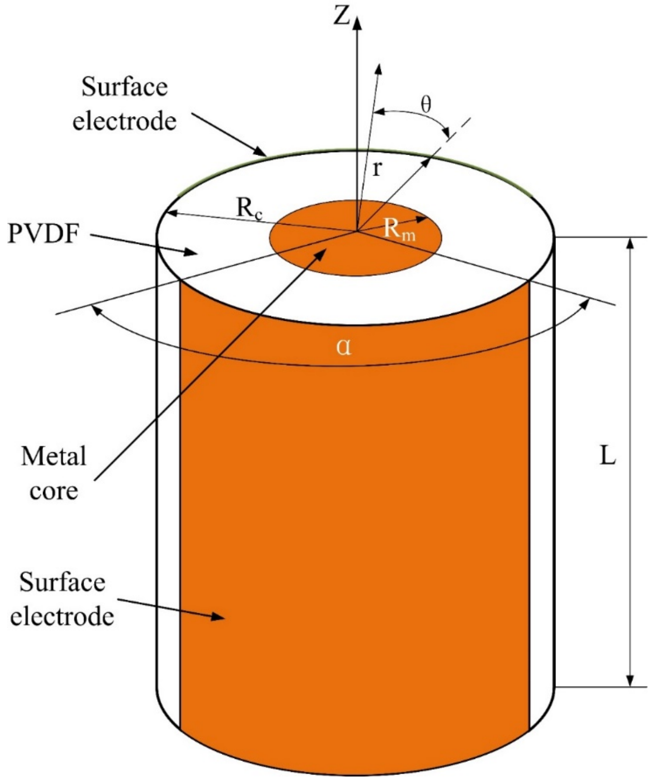

Figure S4 The Schematic diagram of the structure of a SMPF

3. SMPF polarized circuit

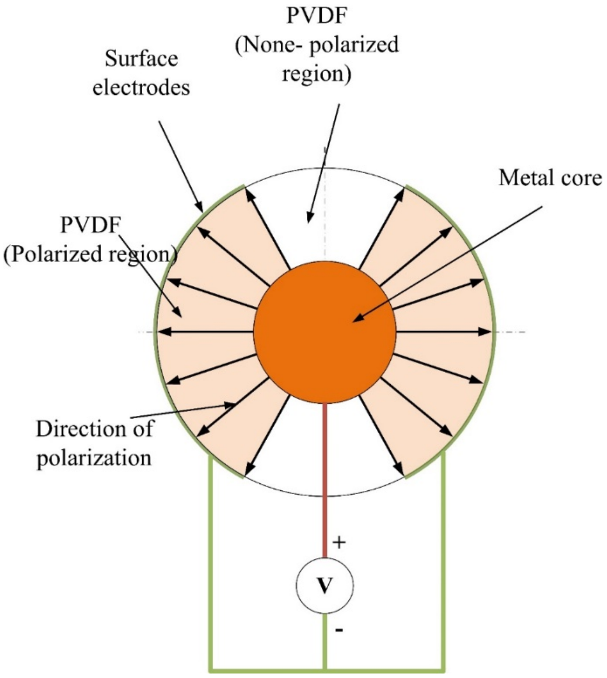

Figure S5 Polarizing circuit of an SMPF

In the polarization process, the polarization circuit of the SMPF is shown in Fig. S5. The metal core was connected to the positive electrode and the two thin metal layers on the surface were connect together to the negative electrode. At this time, in the PVDF region covered by the surface metal thin layer, it could be considered that the electric field was distributed in the radial direction. However, in the PVDF area where the surface was not covered with thin metal layer, it could be considered that there was no electric field distribution. After polarization, it could be considered that the region of the PVDF covered with a thin metal layer was polarized and had piezoelectric properties, and its polarization direction was distributed along the radial direction. In PVDF, the area where the surface is not covered with a thin metal layer is not polarized and does not have piezoelectric properties.

4. Sensing circuit for SMPF

When the SMPF was used as a sensor, the sensing circuit is as shown in Fig. S6. Two thin metal layers on the surface of the SMPF are used as two electrodes of the sensing circuit. When the SMPF cantilever beam was subjected to an external uniform load P, it will produce bending deformation. The angle between P and the electrical boundary of the surface electrode was δ. At this time, that polarization area of the PVDF generated stretching deformation, and due to the piezoelectric effect, charges could be generated on that surface electrode. Because the two

surface electrodes were equal in size, their positions were completely symmetrical. The two surface electrodes produced charges with equal amplitude and opposite polarity. Two surface electrodes were connected to a sensing circuit, and the charges sensed by the circuit was twice the charges generated on one surface electrode.

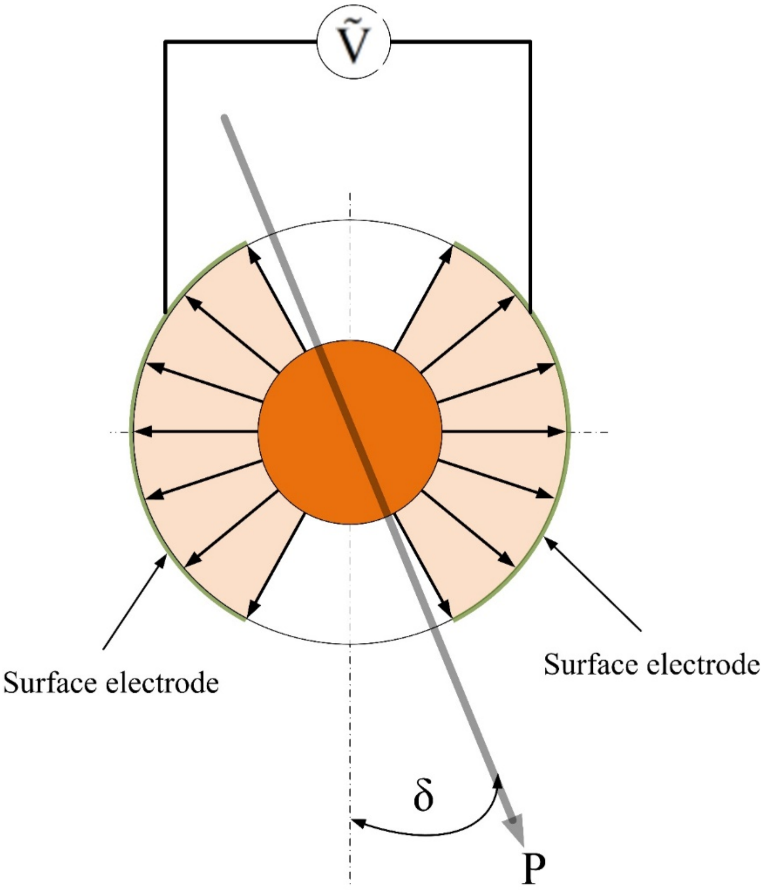

Figure S6 Sensing circuit of a SMPF

5. SMPF bending deformation sensing model

Under linear accelerations, the bionic macula bended transversely due to the inertial force. The SMPF could also produce bending deformation, and the lateral displacement at its free end was U, as shown in Figure S7.

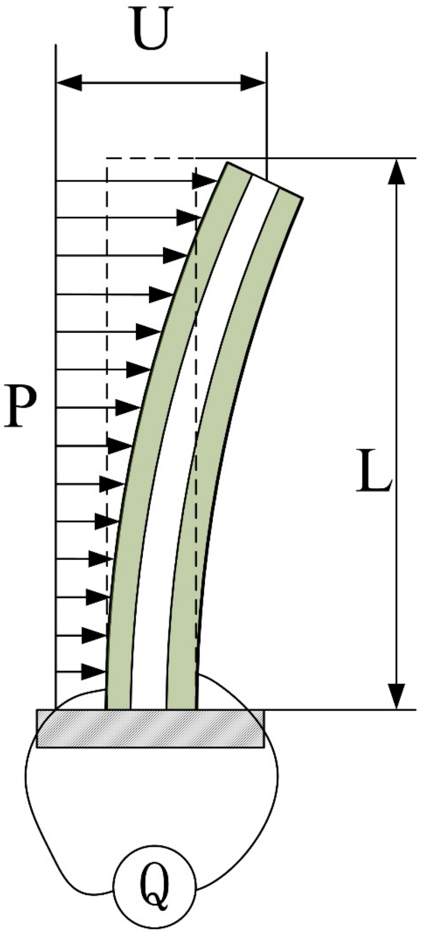

Figure S7 The bending deformation of an SMPF

In the SMPF piezoelectric fiber, the polarized region of the PVDF layer on the surface had piezoelectric effect, and the unpolarized region did not have piezoelectric effect, so the piezoelectric equation of the polarized region in the cylindrical coordinate system could be derived from the first piezoelectric equation as follows:

$$\begin{pmatrix} S_{rr} \\ S_{\theta\theta} \\ S_{zz} \\ S_{r\theta} \\ S_{\theta z} \\ S_{rz} \end{pmatrix} = \begin{pmatrix} s_{33}^E & s_{13}^E & s_{13}^E & 0 & 0 & 0 \\ s_{13}^E & s_{11}^E & s_{12}^E & 0 & 0 & 0 \\ s_{13}^E & s_{12}^E & s_{11}^E & 0 & 0 & 0 \\ 0 & 0 & 0 & s_{55}^E & 0 & 0 \\ 0 & 0 & 0 & 0 & s_{66}^E & 0 \\ 0 & 0 & 0 & 0 & 0 & s_{55}^E \end{pmatrix} \times \begin{pmatrix} T_{rr} \\ T_{\theta\theta} \\ T_{zz} \\ T_{r\theta} \\ T_{\theta z} \\ T_{rz} \end{pmatrix} + \begin{pmatrix} d_{33} & 0 & 0 \\ d_{31} & 0 & 0 \\ d_{31} & 0 & 0 \\ 0 & d_{15} & 0 \\ 0 & 0 & 0 \\ 0 & 0 & d_{15} \end{pmatrix} \times \begin{pmatrix} E_r \\ E_\theta \\ E_z \end{pmatrix} \tag{S1}$$

$$\begin{pmatrix} D_r \\ D_\theta \\ D_z \end{pmatrix} = \begin{pmatrix} d_{33} & d_{31} & d_{31} & 0 & 0 & 0 \\ 0 & 0 & 0 & d_{15} & 0 & 0 \\ 0 & 0 & 0 & 0 & 0 & d_{15} \end{pmatrix} \times \begin{pmatrix} T_{rr} \\ T_{\theta\theta} \\ T_{zz} \\ T_{r\theta} \\ T_{\theta z} \\ T_{rz} \end{pmatrix} + \begin{pmatrix} \varepsilon_{33}^T & 0 & 0 \\ 0 & \varepsilon_{11}^T & 0 \\ 0 & 0 & \varepsilon_{11}^T \end{pmatrix} \times \begin{pmatrix} E_r \\ E_\theta \\ E_z \end{pmatrix} \tag{S2}$$

Where S_{ij} is strain. T_{ij} is the stress. s_{ij}^E denotes the electric field strength. E is elastic

compliance coefficient when the electrical field is zero or a constant; d_{ij} represents the piezoelectric constant. E_i denotes the electric field strength. D_i represents a potential shift. ε_{ij}^{T} denotes stress. T The dielectric constant when the electrical field is zero or a constant.

When the silicone rubber was subjected to bending deformation, the SMPF embedded in it also deformed due to the uniform load acting on the SMPF. Because the length of the SMPF was much larger than its diameter, it can be considered that the radius of curvature of each part of the SMPF in the radial direction was ρ, and the circumferential stress and shear stress of the fiber could be ignored. Therefore, in the piezoelectric equation, the conditions of the stress and electric field could be expressed as follows:

$$S_{rr} = S_{\theta\theta} = S_{r\theta} = S_{\theta z} = S_{rz} = 0 \tag{S3}$$

$$D_{\theta} = D_{z} = 0 \tag{S4}$$

$$E_{r} = 0 \tag{S5}$$

Substituting the Eq. (S3), (S4), and (S5) into the formulas (S1) and (S2), it could be obtained as follows:

$$D_{r} = d_{31}\frac{S_{zz}}{s_{11}^{E}} \tag{S6}$$

At this time, that was

$$\frac{M_c}{E_c I_c} = \frac{M_m}{E_m I_m} = \frac{1}{\rho} \tag{S7}$$

where E_c and E_m are the elastic moduli of the PVDF portion and the metal core in the SMPF, respectively, and I_c and I_m are the moments of inertia of the PVDF portion and the metal core in the SMPF, respectively. Therefore, the inertia bending moment of the whole SMPF could be described as follows:

$$M_c + M_m = M \tag{S8}$$

where M_c and M_m are the bending moments of the PVDF part and the metal core in the SMPF, respectively.

Submitting Eq. (S7) into Eq. (S8) , it could be obtained as follows:

$$M = \frac{M_c}{E_c I_c} + \frac{M_m}{E_m I_m} = \frac{2}{\rho} \tag{S9}$$

Because that

$$E_m = 1/s_m \tag{S10}$$

$$E_c = 1/s_{11}^E \tag{S11}$$

Substituting Eq. (S10) and Eq. (S11) into Eq. (S9), it could be obtained as follows:

$$\frac{1}{\rho} = \frac{M}{E_m I_m + E_c I_c} = \frac{4Ms_m s_{11}^E}{\pi(s_m R_c^4 - s_m R_m^4 + s_{11}^E R_m^4)} \tag{S12}$$

Substituting Eq. (S12) into Eq. (S9), the equivalent bending stiffness of the SMPF could be described as follows:

$$E_c I_c = \frac{1}{4s_{11}^E s_m} \pi(s_m R_c^4 - s_m R_m^4 + s_{11}^E R_m^4) \tag{S13}$$

At this time, the strain at the point(R, θ) on the surface electrode of the SMPF could be calculated according to the following equation.

$$S_{zz}(R,\theta) = \frac{MR\sin\theta}{EI} \quad (R_m < R < R_c) \tag{S14}$$

Substituting the above Eqs into Eq. (S6), the potential shift at this point could be obtained as follows:

$$D_r = d_{31}\frac{MR\sin\theta}{s_{11}^E EI} \quad (R_m < R < R_c) \tag{S15}$$

At this time, the charge on one electrode on the surface of the SMPF could be described as follows:

$$Q = \int_0^L \int_{\frac{\pi-\alpha}{2}}^{\frac{\pi+\alpha}{2}} d_{31}\frac{MR\sin\theta}{s_{11}^E EI} R_c d\theta dz = \frac{2Ld_{31}MR_c \sin\frac{\alpha}{2}}{S_{11}^E EI} \tag{S16}$$

When the BU was subjected to impact vibrations, the impact vibration function at the root of SMPF is:

$$u(t) = U_0\left(1 - e^{-\tau t}\right)\left(xT < t < xT + \frac{T}{20}\right) \tag{S17}$$

Where U_0 is the initial amplitude and τ is the time constant, then the equivalent mean load could be expressed as follows:

$$P = U_0 \tau^2 \mathrm{e}^{-\tau t} \tag{S18}$$

The transverse deformation of the SMPF could be obtained as follows:

$$\Delta y = -\frac{\mathrm{p}z^3}{24EI}(z^2 - 4lz + 6l^2) \tag{S19}$$

At this time, the bending moment of SMPF under the action of uniform load P is

$$M = \int_z^L \mathrm{p}z dz = \frac{1}{2}\mathrm{p}\left(L^2 - z^2\right) \tag{S20}$$

At the end of the SMPF, Z = L, the transverse deformation is

$$U = U_0 \tau^2 \mathrm{e}^{-\tau t} \frac{-L^4}{8EI} \tag{S21}$$

where E and I represent the elastic modulus and the moment of inertia of the material respectively.

The bending moment of SMPF could be described as follows:

$$M = \frac{1}{2} P L^2 \tag{S22}$$

The relationship between the output charges of the SMPF and the lateral deformation at its end could be derived from Eq. (S16), (S21), and (S22) as follows:

$$Q = \frac{8 d_{31} R_c}{S_{11}^E L} \sin\frac{\alpha}{2} \times U_0 \tau^2 \mathrm{e}^{-\tau t} \frac{-L^4}{8EI} \tag{S23}$$

The Eq. (S21) indicated that the lateral displacement of the end of the SMPF was linear with the amplitude of the impact vibration, and the Eq. (23) confirmed that the output charges of the SMPF was linearly related to the amplitude of the impact vibration.

6. Sensing signal of SMPF sensors when subjected to shock vibration

When the BU is subjected to impact vibrations, as shown in the Fig. s8

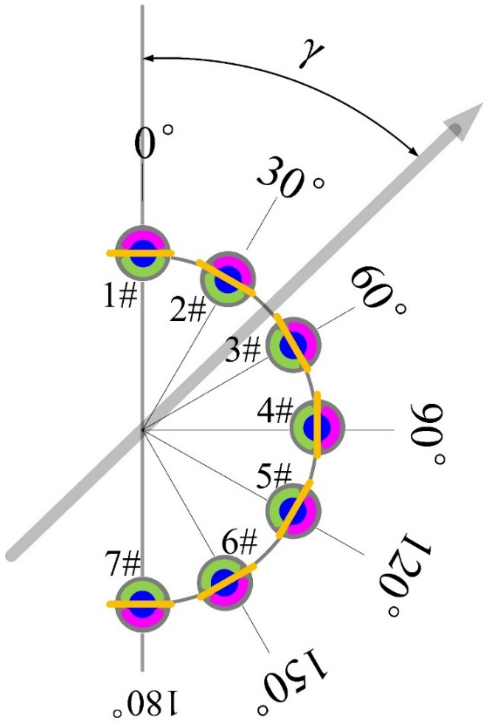

Figure S8 The BU subjected to an impact vibration

The sensing signal of the SMPF sensors is as follows:

$$Q_i = A_0 B_0 \cos\left|(i-1)\times\frac{\pi}{6}-\gamma\right| \tag{S24}$$

The output charges of the 7 SMPF sensors can be described as follows:

$$\begin{cases} Q_1 = A_0 B_0 \cos(\gamma) \\ Q_2 = A_0 B_0 \cos(\gamma-\frac{\pi}{6}) \\ Q_3 = A_0 B_0 \cos(\frac{\pi}{3}-\gamma) \\ Q_4 = A_0 B_0 \cos(\frac{\pi}{2}-\gamma) \\ Q_5 = A_0 B_0 \cos(\frac{2\pi}{3}-\gamma) \\ Q_6 = A_0 B_0 \cos(\frac{5\pi}{6}-\gamma) \\ Q_7 = A_0 B_0 \cos(\pi-\gamma) \end{cases} \tag{S25}$$

REFERENCES

10. Jaeger, R.; Haslwanter, T. Otolith responses to dynamical stimuli: Results of a numerical investigation. *Biol. Cybern.* **2004**, *90*, 165–175, doi:10.1007/s00422-003-0456-0.

19. Curthoys, I.S. Concepts and physiological aspects of the otolith organ in relation to electrical stimulation. *Audiol. Neurotol.* **2020**, *25*, 25–34, doi:10.1159/000502712.

32. Telban, R.J.; Cardullo, F. Motion cueing algorithm development: Human-centered linear and nonlinear approaches. *NASA TechReport* **2005**, CR-2005-213747.
